# Automated multi-model deep neural network for sleep stage scoring with unfiltered clinical data

**DOI:** 10.1007/s11325-019-02008-w

**Published:** 2020-01-14

**Authors:** Xiaoqing Zhang, Mingkai Xu, Yanru Li, Minmin Su, Ziyao Xu, Chunyan Wang, Dan Kang, Hongguang Li, Xin Mu, Xiu Ding, Wen Xu, Xingjun Wang, Demin Han

**Affiliations:** 1 Beijing Tongren Hospital, Capital Medical University, Beijing, 100730 People’s Republic of China; 2 Obstructive Sleep Apnea-Hypopnea Syndrome Clinical Diagnosis and Therapy and Research Centre, Capital Medical University, Beijing, 100730 People’s Republic of China; 3 Key Laboratory of Otolaryngology Head and Neck Surgery, Ministry of Education, Capital Medical University, Beijing, 100730 People’s Republic of China; 4 Department of Electronic Engineering, Tsinghua Shenzhen International Graduate School, Tsinghua University, Shenzhen, China

**Keywords:** Polysomnography (PSG), Obstructive sleep apnea (OSA), Sleep staging, Deep learning

## Abstract

**Purpose:**

To develop an automated framework for sleep stage scoring from PSG via a deep neural network.

**Methods:**

An automated deep neural network was proposed by using a multi-model integration strategy with multiple signal channels as input. All of the data were collected from one single medical center from July 2017 to April 2019. Model performance was evaluated by overall classification accuracy, precision, recall, weighted F1 score, and Cohen’s Kappa.

**Results:**

Two hundred ninety-four sleep studies were included in this study; 122 composed the training dataset, 20 composed the validation dataset, and 152 were used in the testing dataset. The network achieved human-level annotation performance with an average accuracy of 0.8181, weighted F1 score of 0.8150, and Cohen’s Kappa of 0.7276. Top-2 accuracy (the proportion of test samples for which the true label is among the two most probable labels given by the model) was significantly improved compared to the overall classification accuracy, with the average being 0.9602. The number of arousals affected the model’s performance.

**Conclusion:**

This research provides a robust and reliable model with the inter-rater agreement nearing that of human experts. Determining the most appropriate evaluation parameters for sleep staging is a direction for future research.

**Electronic supplementary material:**

The online version of this article (10.1007/s11325-019-02008-w) contains supplementary material, which is available to authorized users.

## Introduction

Obstructive sleep apnea (OSA) is a disease characterized by recurrent partial or complete upper airway collapse obstruction during sleep, which can cause repeated apnea and hypopnea, often accompanied by hypoxemia, sleep disturbance, hypertension, coronary heart disease, and diabetes. OSA is the source of various cardiovascular and cerebrovascular diseases, endocrine diseases, and throat diseases. Epidemiological studies revealed that 936 million people worldwide suffer from moderate to severe OSA, and the number of people affected in China is among the highest in the world, causing a substantial social and economic burden [1]. Furthermore, studies suggest that 80%–90% of cases remain undiagnosed [1]. Therefore, it is crucial to improve the efficacy of diagnosis of OSA.

The diagnosis of OSA relies on overnight polysomnography (PSG) and manual data analysis in sleep laboratories. Sleep stage scoring criteria are standardized and follow the latest updates from the American Academy of Sleep Medicine (AASM) [2]. However, sleep stage scoring still relies on manual interpretation from skillful technicians. Thus, the traditional PSG scoring is time consuming [3], and therefore an automated sleep staging system would assist sleep experts and provide great clinical utility.

Deep learning, as a field in machine learning research, has undergone an expansion of its application space in recent years, promoting rapid analysis of complex image data; assisting in the screening, diagnosis, and follow-up of related diseases; and significantly shortening the diagnostic time with limited medical resources. Electroencephalography (EEG) is a nonstationary signal and has a low signal-to-noise ratio (SNR), but new ways are needed to improve EEG processing to achieve better generalization capabilities and more flexible application. Recently, deep learning (DL) has shown great promise in identifying EEG signals due to its capacity to learn good feature representations from raw data. The majority of studies tackling this issue adopt convolutional neural networks (CNNs), recurrent neural networks (RNNs), or a CNN + RNN as the neural network architecture for sleep staging [4], and an accuracy rate greater than 87% has been reached [4].

In clinical settings, the scoring of sleep staging is complicated because the PSG processing could be confronted with challenging conditions, such as electrode shedding, signal artifacts, and noise. In this study, we use unfiltered clinical data and deep learning to develop automated analysis algorithms and validate them and to explore the scope of application in clinical practice.

## Materials and methods

This retrospective study was approved by the institutional review board of Beijing Tongren Hospital (TRECKY2017–032).

### Subjects

All of the subjects were 18–70 years of age and had a history of habitual snoring. All of the subjects underwent overnight PSG in the sleep medicine center, Beijing Tongren Hospital from July 2017 to April 2019. Patient demographics were obtained for all of the subjects. The training dataset, validation dataset, and testing dataset were independent of one another based on inspection time. Patients less than 14 years old or had a time in bed (TIB) less than 4 h were excluded. Details are summarized in Table 1.

**Table 1:** Demographics and characteristics of datasets

	Training	Validation	Testing	*P* value
Number of participants/epochs	92/93,788	21/20,845	152/150103	
Normal	13/13081	3/2958	23/22741	
Mild OSA	19/19612	4/3976	23/22580	
Moderate OSA	17/17163	4/4262	29/27774	
Severe OSA	43/43932	10/9649	77/77008	
Sex (male: female)	64:28	16:4	129:33	> 0.05
Age (median, range)	42.5 (19–68)	47.5 (22–57)	38.0 (79–61)	< 0.05*
BMI (kg/m^2^) (median, range)	25.95 (16.1–38.4)	27.65 (18.8–34.0)	26.55 (13.8–46.3)	> 0.05
TST (min) (median, range)	423.10 (200.5–577.6)	436.10 (285.5–510.4)	426.85 (92.0–578.5)	> 0.05
AHI (median, range)				
Normal	1.8 (0.5–4.2)	1.2 (0.6–2.2)	1.4 (0.2–4.9)	> 0.05
Mild OSA	11.1 (5.4–13.5)	9.7 (7.7–14.3)	9.1 (5.4–14.1)	> 0.05
Moderate OSA	19.9 (15.3–29.2)	18.45 (15.1–29.7)	23.7 (15.1–28.8)	> 0.05
Severe OSA	51.8 (30.6–105.3)	66.6 (37.3–97.7)	56.9 (30.9–112.4)	> 0.05
Sleep stage (*n*, %)				
W	16,201	2339	26,112	
N1	14,839	3574	24,489	
N2	47,889	10,744	73,395	
N3	1881	648	3289	
R	12,978	3540	22,818	
Minimum SpO_2_ (%) (median, range)	85 (51–96)	83 (37–94)	83 (35–95)	> 0.05
Number of arousals (median, range)	79.5 (1–592)	97 (7–528)	79.5 (0–692)	> 0.05

*BMI* body mass index, *TST* total sleep time, *AHI* apnea–hypopnea index, *SpO*_*2*_ pulse oxygen saturation

### Polysomnography

Overnight, PSG was performed on all of the participants by the Philips Respironics G3 sleep diagnostic system, including a 2-channel electroencephalography (EEG) (C3/A2, C4/A1), 2-channel electrooculography (EOG), anterior tibial electromyogram (EMG), electrocardiogram (ECG), 2-channel airflow measurement with nasal cannula pressure, recording of respiratory (thoracic and abdominal) movements, and pulse oximetry for oxygen saturation (SpO_2_). All of the ECG and EOG channels were captured at a 200 Hz sampling frequency and displayed with a 0.3–35 Hz band-pass filter. Anterior tibial EMG had a sampling rate of 200 Hz, and the band-pass filter was 10–100 Hz.

Two highly trained, experienced (more than 10 years) PSG technologists scored sleep stages and respiratory events in 30 s epoch in accordance with the American Association of Sleep Medicine (AASM 2012) guidelines [5]. The apnea–hypopnea index (AHI) was defined as the number of apnea and hypopnea events per hour of sleep and was used to indicate the severity of sleep apnea (normal: AHI < 5; mild OSA, 5 ≤ AHI < 15; moderate OSA, 15 ≤ AHI < 30; severe OSA, AHI ≥ 30).

### Data processing

According to the AASM standard, the central band of the EEG signal is concentrated below 35 Hz, while the sampling rate is 200 Hz. Instead of getting more information from the excessive sampling frequency, we only get high-frequency noise. Therefore, we first filtered the signal at 66 Hz and then downsampled the signal sampling frequency to 66 Hz (which is one-third of the original sampling frequency) to remove the influence of high-frequency noise while ensuring that no spectral aliasing occurs and to reduce the amount of data.

**Fig. 1:**
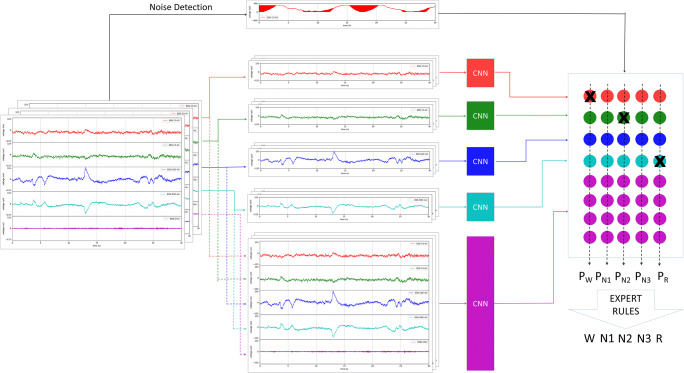
Overall architecture of our method. The left side shows the input signals, consisting of 5 channels: EEG C3/A2, EEG C4/A1, 2-channel EOG, and EMG. The 5-channel signal is divided into 5 groups, as shown in the middle of the figure: Groups 1 to 4 are EEG C3/A2, EEG C4/A1, and 2-channel EOG, respectively, and the fifth group consists of all 5 input signals. Then each group of signals was feed into a CNN model for training and prediction. At the same time, a noise detection algorithm detected the noise in each group. The right part of the illustration shows the integration, and the colored nodes represent integration weights corresponding to different CNN models. We take the weighted-average as each stage’s probability. Notice that the “X” on the weight means that this weight is reset to zero due to noise. After integration, the output prediction was modified by expert-defined rules

Considering the sleep continuity, the staging of each epoch may correlate with the previous and subsequent epochs. A 90-s window (3 epochs) to redivide the signal was applied with a stride of 30 s, which means that newly divided epoch’s length was three times the original length. The newly divided epoch took the stage label of the original 30 s epoch as its label (Fig. 1).

### Neural network

(The details are in the supplementary materials)

### Training

(The details are in the supplementary materials)

### Noise detection

(The details are in the supplementary materials)

### Expert rules

The REM stage is exceptional in EEG staging. Although the REM stage has specific characteristics, rapid eye movements do not occur within every 30-s epochs. However, it is quite difficult for the model to determine whether these epochs with no rapid eye movements are in the REM stage because it relies on prior knowledge of the current stage. Therefore, we checked each epoch’s next eight epochs: if there was a REM stage epoch, we forcibly converted this epoch to the REM stage, thus ensuring the continuity of the REM period.

### Model architecture

**Fig. 2:**
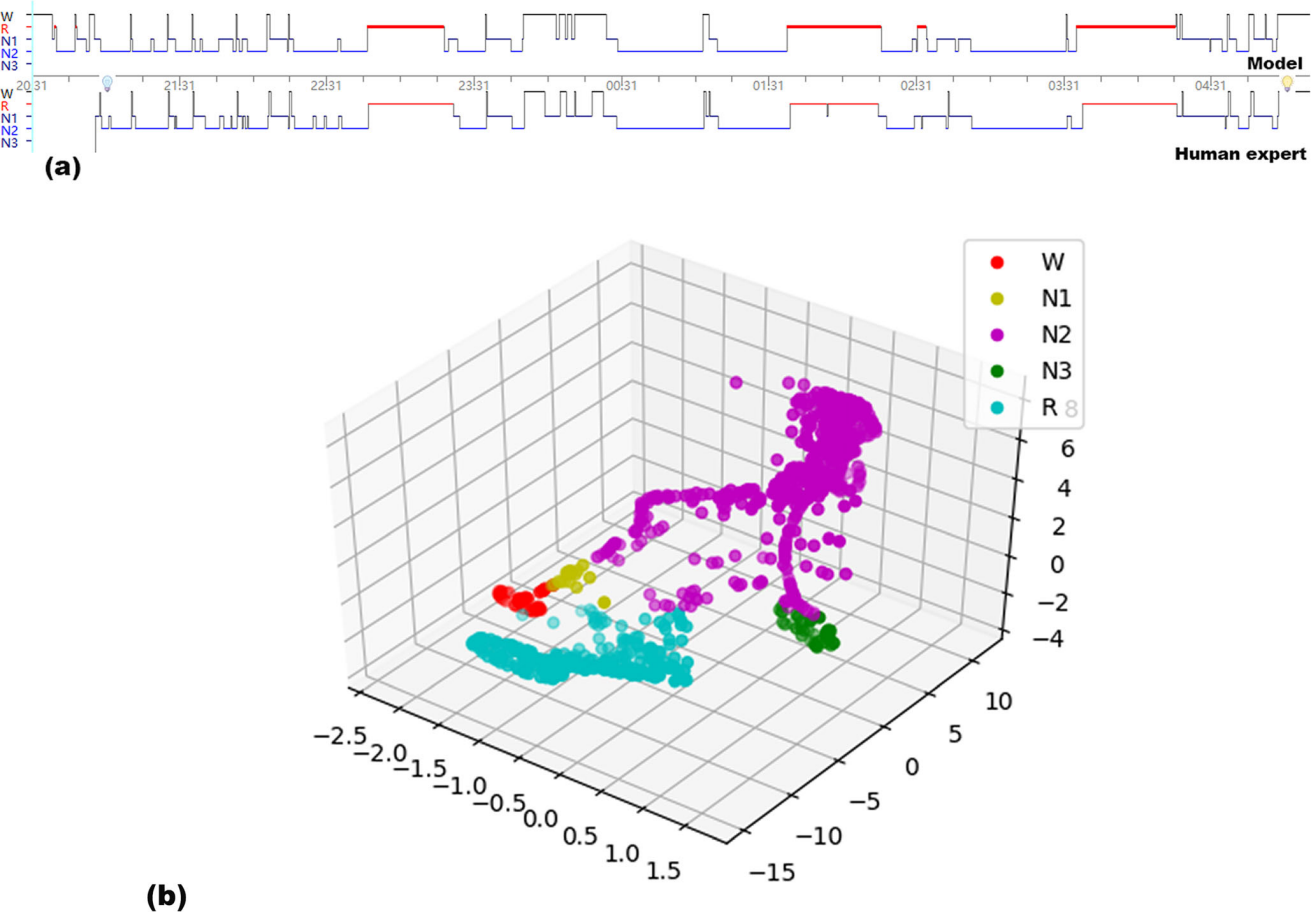
(**a**) Example of an overnight PSG record scored by the model vs. human expert. (**b**) t-SNE for the last hidden layer of the CNN. Each differently colored point indicates a sleep stage scored by the model, suggesting that the model can discriminate different sleep stages well

The overall algorithm framework is shown in Fig. 2. After preprocessing, the signal was input into the corresponding CNN model and the real-time noise detection module. The outputs of multiple models were integrated while setting the weights of the falloff signal models to zero. Then the model modified the integrated prediction of multiple models by expert-defined rules to get the final prediction.

### Model evaluation and statistical analysis

The performance of sleep stage prediction was measured by overall classification accuracy, precision, recall, weighted F1 score, and Cohen’s Kappa. Top-2 accuracy was applied, which means that the two most probable predictions for the model prediction were considered “correct.”

The confusion matrix was applied to the visualization of the performance of algorithms.

Statistical analysis was performed using SPSS 25 software (SPSS Inc., Chicago, IL). The Shapiro–Wilk test was used to verify normal value distribution. Differences in variables were analyzed by Student’s t-test or Mann–Whitney U test. All of the *P* values were 2-sided, and *P* values less than 0.05 were considered to be significant.

### Cross dataset experiments

To further evaluate the performance of our method, we evaluated it on a public dataset named Sleep-EDF. In order to compare our method with others, we used the 2013 version, which contains two sets of subjects from two studies: age effect in healthy subjects (SC) and Temazepam effects on sleep (ST). Two PSGs of about 20 h each were recorded during two subsequent day–night periods at the subjects’ homes. Well-trained technicians manually scored corresponding hypnograms (sleep patterns) according to the Rechtschaffen and Kales manual. As AASM recommends, N3 and N4 of the sleep-EDF dataset were merged in this study. Twenty in-bed SC subjects (age 28.7 ± 2.9) were used. Each PSG recording contained 2 scalp-EEG signals (Fpz-Cz and Pz-Cz), 1 EOG (horizontal), 1 EMG, and 1 oral–nasal respiration signal. All EEG and EOG had the same sampling rate of 100 Hz. The SC dataset was divided into five folds for training and independent validation.

## Result

### Population characteristics

The numbers of PSG subjects in the training dataset, the validation dataset, and the testing dataset were 122, 20, and 152, respectively. Of the three datasets, males accounted for the vast majority. No significant differences were detected in sex, BMI, total sleep time, AHI, sleep stage distribution, minimum SpO2, and number of arousals, suggesting that the samples in the three datasets were homogeneous. The only significant difference was detected in age.

### Comparative study to choose the best algorithm

To select the most appropriate model, comparative studies were conducted to evaluate the same testing dataset; the results are summarized in Table 2. Models with neither the 3-epoch splice, expert rules nor noise detection resulted in lower evaluation parameters (Fig. 2).

**Table 2:** Model performance with different training algorithms

Training algorithm	Macro-accuracy	Weighted F1 score	Cohen’s Kappa
Without the 3-epoch splice	0.8034	0.7885	0.7044
Without noise detection	0.8050	0.7996	0.7105
Without expert rules	0.8173	0.8115	0.7266
The proposed model	0.8181	0.8150	0.7276

*AHI* apnea–hypopnea index

### Model performance

**Fig. 3:**
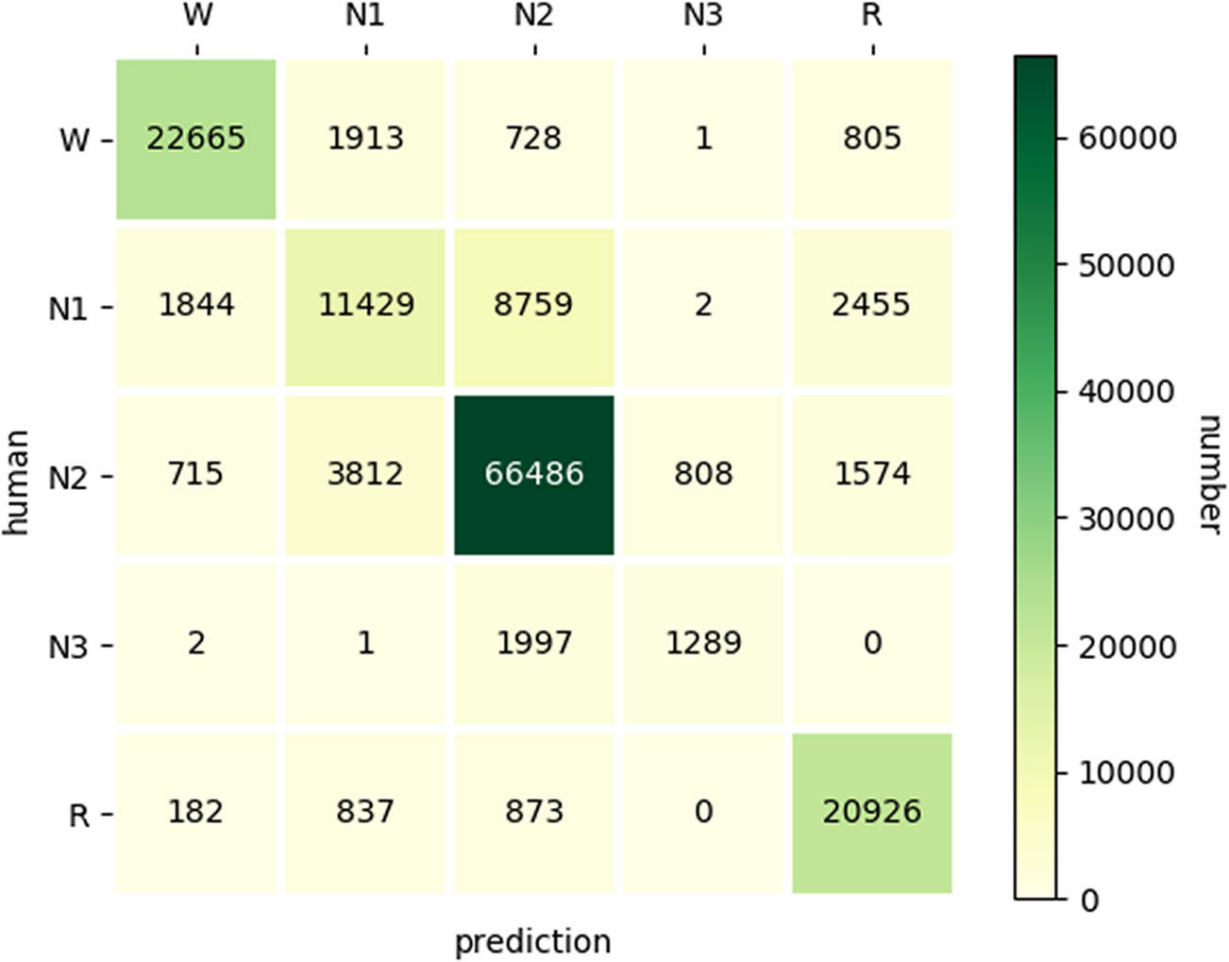
Confusion matrix for the predicted sleep stage, displaying the agreement with expert scores.The vertical rows represent the sleep staging scored by the human expert, while the horizontal rows are the predictions for the same epoch of the testing dataset. The diagonal numbers are the epochs for which the prediction of the model matches the human expert at each sleep stage. The model possesses higher consistency for W, N2, and R identification

Table 3 presents more detailed results of the model described above. The average predicted TST was 410.18 min, compared 426.85 min calculated by human experts. The population of the testing dataset was divided into four groups according to the degree of AHI. The normal population received the highest accuracy and the highest weighed F1 score. The confusion matrix demonstrated that the most appropriate model after comparative studies possesses higher consistency for W, N2, and R identification but has poor performance for N1 and N3 (Fig. 3). Moreover, the F1 score and Cohen’s Kappa indicated moderate to strong inter-rater agreement between the model performance and human experts on weighted average performance for both classification by AHI and by sleep stage (Table 4).

**Table 3:** Model performance on testing dataset according to AHI

Testing dataset	Macro-accuracy	Weighted-F1 score	Cohen’s Kappa
Normal	0.8361	0.8277	0.7560
Mild OSA	0.8265	0.8221	0.7433
Moderate OSA	0.8222	0.8153	0.7288
Severe OSA	0.8088	0.7981	0.7124
Weighted average	0.8181	0.8150	0.7276

*AHI* apnea–hypopnea index

**Table 4:** Model performance on sleep staging of testing dataset

Sleep staging	Precision	Recall	F1 score	Number of epochs
W	0.8920	0.8680	0.8799	25,408
N1	0.6352	0.4667	0.5381	17,992
N2	0.8433	0.9059	0.8734	78,843
N3	0.6138	0.3919	0.4784	2100
R	0.8123	0.9171	0.8615	25,760
Weighted average	0.8181	0.8150	0.7276	150,103

### Top-2 accuracy on sleep stage scoring

Since there is inter-rater variability between technicians in sleep staging, in this study, we introduce an evaluation index called the top-2 accuracy, defined as the proportion of test samples for which the correct label is among the two most probable labels given by the model. The neural network is an appropriate method mathematically; in classification tasks, it judges the similarity between the input sample and the data distribution corresponding to each label, scores the similarities, and normalizes them into probability. If the correct label of a sample is among the two most probable labels given by the neural network, we consider this sample as an exact sample in the context of the top-2 accuracy (Table 5).

**Table 5:** Model performance on testing dataset

Testing dataset	Top-1 macro-accuracy	Top-2 macro-accuracy	Average increase rate
Normal	0.8341	0.9611	0.1270
Mild	0.8292	0.9698	0.1407
Moderate	0.8228	0.9512	0.1285
Severe	0.8088	0.9619	0.1531
The average performance	0.8184	0.9602	0.1419

AHI apnea–hypopnea index

Table 6 presents the model’s predictions for each epoch with the two most probable predictions. For example, the difference between N1 and W in EEG is sometimes not obvious, and different technicians will have different judgments. The model in this paper has a judgment ability close to that of a human technician for the confusing EEG. The most striking result was that when the model output N2, the second possible sleep stage was N1 stages accounted for 66.79%. Similarly, the second possible sleep stage was N2 when the model outputs N3 in most instances. Since the REM stages can be divided into the phasic mode and tonic mode, the second identification of the model distributed in the W stages, N1 stages, and N2 stages, accounts for the majority.

**Table 6:** Distribution of sleep staging with two most significant predicted probabilities for each epoch (without expert rules)

Number of epochs (%*)		The second largest probability of prediction				
		W	N1	N2	N3	R
Maximum probability of prediction (model output)	W	–	11,984 (47.17%)	629 (2.47%)	15 (0.06%)	2119 (8.33%)
	N1	3994 (22.20%)	–	6294 (34.98%)	0 (0%)	2795 (15.53%)
	N2	1030 (13.06%)	52,656 (66.79%)	–	11,347 (14.39%)	1392 (9.1 × 10^−4^%)
	N3	2 (0.09%)	0	1289 (61.38%)	–	0
	R	1265 (6.91%)	14,886 (57.79%)	3577 (13.89%)	0 (0%)	–

*Proportion of the same epoch of the testing dataset

**Fig. 4:**
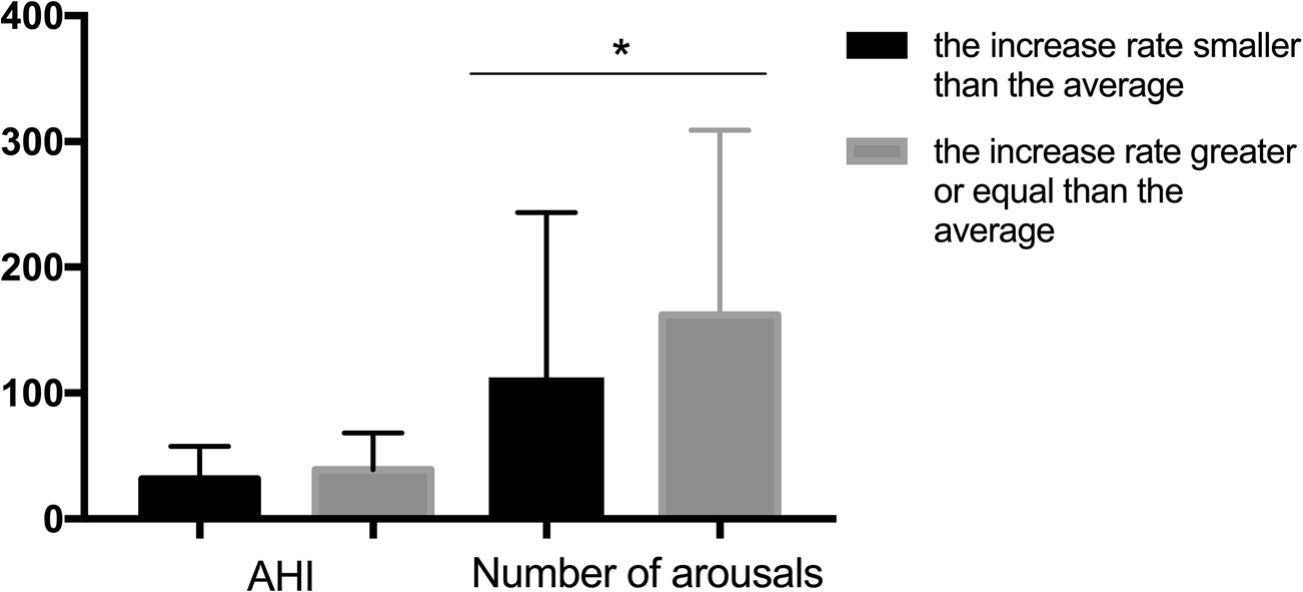
According to the growth rate of accuracy, there was no statistical difference in AHI between the two groups, but the number of arousals demonstrated significant differences (p<0.01). AHI=Apnea–Hypopnea Index

In order to explore the reasons behind this, we try to divide the data in the testing dataset into two groups: the increased rate of accuracy greater or equal to the average increase rate (0.1419) and the increase rate smaller than the average. Statistical analysis showed that the greater the number of arousals, the higher the top-2 accuracy. In other words, the number of arousals affected the model’s performance on sleep staging (Fig. 4).

### Evaluation of cross dataset experiments

Studies comparing the proposed model to other methods on sleep-EDF are summarized in Table 7. The model showed improvement in all metrics. Compared with the testing dataset results from our clinical center, the per-class F1 of the N3 stages was significantly improved. However, the per-class F1 of the N1 period was still lower than that of other sleep stages, which is consistent with the findings of other studies [6–8].

**Table 7:** Comparation of other methods to the proposed method

Method	Channel	Acc	Macro-average F1	Per-class F1					Cohen’s Kappa
				W	N1	N2	N3	R	
Supratak A et al. [7]	Fpz-Cz	0.820	0.769	0.847	0.466	0.859	0.848	0.824	0.76
Supratak A et al. [7]	Pz-Oz	0.798	0.731	0.881	0.370	0.827	0.773	0.803	0.72
Tsinalis O et al. [6]	Fpz-Cz	0.789	0.737	0.716	0.370	0.846	0.840	0.814	0.65
Sun Y, et al. [8]	Pz-Oz	0.810	0.736	0.856	0.249	0.889	0.792	0.863	0.73
The proposed model	All	0.836	0.781	0.864	0.498	0.887	0.845	0.816	0.77

*Acc* accuracy

## Discussion

In this study, the model performed robustly under different levels of AHI and performed slightly better in the healthy population than in patients with severe OSA. As the AHI increased, the accuracy and F1 values gradually decreased. In patients with severe OSA, the lowest value is considered to be related to fragmented sleep, and the EEG is relatively complicated. Cohen’s Kappa was to evaluate the inter-rater variability between the model and the technician’s scoring. The literature suggests that there is inter-rater variability between different human technicians, and both N1 and N3 are relatively low, ranging from 20% to70% [9–12]. The average Cohen’s Kappa of this study was 0.7276, indicating a substantial agreement with human technicians. Similar to the previous pieces of literature, the model displayed a low consistency in the N1 and N3 stages. Such a result considers that the waveform characteristics of the low amplitude in the N1 stage are not prominent, and the model may confuse N1 with N2 during scoring (like when the EEG is not typical and thus a technician confuses N1 and N2). However, the agreement of N3 is weak due to the high proportion of OSA patients in the training dataset, which may lead to the number of N3 periods being inadequate, accounting for only 2% of the total number of epochs. In clinical practice, the number of sleep stages in clinical data is imbalanced. Compared with healthy people, sleep fragmentation in OSA patients has more W and N1 stages and fewer N3 stages. In this study, because the unfiltered data was closer to the clinical situation, the imbalanced sample categories will result in too few features and too diminutive a sample size to extract the data pattern, or in over-fitting problems because of limited samples. For the test of the public dataset, the metrics were significantly improved in N3 stages

To determine the final model architecture, this study conducted a comparative study on the same testing dataset. In the clinical PSG, there may be a decrease in signal quality due to sweating, intolerance to the environment, limb movement, and so forth. Therefore, the model design of this study considers the possibility of abnormal signal acquisition during overnight sleep PSG. Second, since there are transitional rules associated with the sleep staging, Markov models, CNNs, and RNNs have been used in recognition of sleep EEG in recent years [13–17]. This research innovatively applied the method of three-epoch splicing to simulate the technician recognition of EEG, so that if there is an epoch with atypical or severe interference, technicians could refer to the previous and following epochs of the EEG. Another innovation in this study is the addition of expert rules. In clinical practice, the identification of REM stages mainly includes rapid eye movement, low-tension diaphragmatic electromyography, sawtooth waves, and transient myoelectric activity. The tonic mode of REM sleep should not have any apparent ocular activity so that the model does not make erroneous judgments. Expert rules can substantially avoid erroneous judgments.

To explore the analysis process of the model, this study innovatively introduced the concept of top-2 accuracy. As a result, the overall accuracy was dramatically improved. Through the analysis of the predicted value of the second probability of the model, this study finds that the model will have a certain degree of confusion when distinguishing between the W and N1 stages, between the N1 and N2 stages, and between N2 and N3 stages; this is consistent with the most common differences in sleep scoring by human experts [18]. A previous study pointed out that the definition of divergence and the K complex wave lacks specificity and is related to the existence of spindle wave identification [19]. Since the lack of a clear “absolute truth value” for sleep stage scoring, the substantial increase in top-2 accuracy indicates that the model output is reasonable. This study found that arousal affects the accuracy of sleep staging; this may be due to the number of arousals being positively correlated with the number of N1 stages [20]. Moreover, the model performance of N1 is lower than that of other sleep stages. This study proposes a future direction for the evaluation of deep learning algorithms by analyzing the top two rankings of maximum probability values for sleep staging. For sleep staging, which relies on manual scoring and must consider inter-rater variability, it is worthwhile to study which parameters are chosen to evaluate model performance. Three classifications (awakening, NREM, and REM) or four classifications (awakening, shallow sleep (N1 + N2), deep sleep (N3), and REM)) make sense in clinical practice.

There are some limitations to this study. First, the clinical data of this study is imbalanced, and the number of N3 stages in this study is small. Compared with other studies, the recognition of N3 is lower. Second, the clinical dataset used in this study was derived from retrospective data of a single center, lacking analysis of homogeneity with the published dataset sleep-EDF. Additionally, the study applied independent and homogeneous training sets and testing sets without cross-validation, and thus there may be deficiencies in the assessment of the generalization capabilities of the model.

## Conclusion

In conclusion, this research provides a robust and reliable model in which the inter-rater agreement nears that of human experts. In future research, it is essential to address the abovementioned limitations, explore the evaluation criteria for neural network models, and develop a lightweight version of the model to make it work in wearable devices and smart devices. Eventually, this work can have a positive impact on population health and healthcare expenditures.

## Electronic supplementary material

ESM 1 Architecture of the neural network. The illustration on the left is the overall architecture of the model, consisting of several groups of convolution blocks (conv blocks) and transition blocks (trans-blocks) and a flatten layer followed by a Dense layer to predict the sleep stages. The illustration on the right top is the architecture of the trans-block. It consists of two convolution layers, each followed by a batch normalization layer and a ReLU activation layer. In addition, a short connection concatenates the input and the output of the two convolution layers, and an average pooling layer halves the concatenated features. The right-bottom illustration is the architecture of the trans-block, which consists of two convolution layers with strides of 2 and 1; this block reduces the feature length by half. (PNG 31 kb)

High-Resolution Image (TIF 4607 kb)

ESM 2 (DOCX 14 kb)
