# Supplementary figures and images for: Automated multi-model deep neural network for sleep stage scoring with unfiltered clinical data
Source: Sleep Breath. 2020 Jan 14;24(2):581–90. doi: 10.1007/s11325-019-02008-w (PMC7289784; doi:10.1007/s11325-019-02008-w)

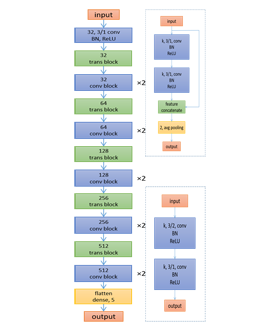

Supplement: Supplementary file 1 — Architecture of the neural network. The illustration on the left is the overall architecture of the model, consisting of several groups of convolution blocks (conv blocks) and transition blocks (trans-blocks) and a flatten layer followed by a Dense layer to predict the sleep stages. The illustration on the right top is the architecture of the trans-block. It consists of two convolution layers, each followed by a batch normalization layer and a ReLU activation layer. In addition, a short connection concatenates the input and the output of the two convolution layers, and an average pooling layer halves the concatenated features. The right-bottom illustration is the architecture of the trans-block, which consists of two convolution layers with strides of 2 and 1; this block reduces the feature length by half. (PNG 31 kb) [file 11325_2019_2008_Fig5_ESM.png]
