# Supplementary material for: Automated multi-model deep neural network for sleep stage scoring with unfiltered clinical data
Source: Sleep Breath. 2020 Jan 14;24(2):581–90. doi: 10.1007/s11325-019-02008-w (PMC7289784; doi:10.1007/s11325-019-02008-w)
Supplement: Supplementary file 3 — (DOCX 14 kb) [file 11325_2019_2008_MOESM2_ESM.docx]

**Supplementary Material**

Neural Network

A convolution neural network (CNN) was used to classify the sleep stage of the signals. The CNN model consisted of two main modules: a convolution block and a transition block. The convolution block convolved the input features, extracted the feature representation, and output the convolved feature map (Supplementary Material_Fig1). As shown in Supplementary Material_Fig1, our convolution block consists of two convolution layers, each followed by a batch normalization layer and a ReLU activation layer. The size of the kernel of both two convolution layers was 3, with a stride of 1. Then the extracted feature was concatenated with the input of the convolution block in the feature dimension, followed by an average pooling layer with a kernel size of 2 and stride of 2. Transition block also had two convolution layers: the stride of the first layer was 2, and the number of kernels for the two layers was twice the number of input channels. The function of the transition block was to halve the length of the feature tensor in the feature dimension and double the width of the feature tensor in the channel dimension, which is critical in deep neural network design (Supplementary Material_Fig1).

Our overall model architecture consisted of the above-mentioned convolution block and transition block. After the feature was extracted by a convolution layer with a kernel size of 3, it was successively passed through modules containing one reduction block and two convolution blocks; this was repeated five times. The length of the feature reduced continuously while the width of the feature (i.e., the number of channels) increased continuously. The last layer of the model was a dense layer (fully connected layer) followed by a Softmax layer.

Training

Each convolutional layer was initialized with He_Normal and regularized with L2 regularization (parameter 1e-4). Each convolutional layer or pooling layer was padded. The training optimizer was Adam (beta_1 = 0.9, beta_2 = 0.999, decay = 0), initialized with a learning rate of 1e-3. The learning rate was reduced to one-tenth of the previous rate whenever loss did not decrease in two epochs; the minimum limit of the learning rate was 1e-6. The model was trained batch by batch with a batch size of 32. An early stopping callback on the validation loss with the patience of 5 epochs was used to stop the training process when no improvements were detected. We trained the model on an NVIDIA RTX2070 GPU.

Noise Detection

An electrode may fall off due to the patient sweating and turning over during the overnight PSG. A sleep expert, when judging sleep stage during the fall-off period, would ignore the signal channel that falls off and concentrate on the normal signal channels. Therefore, we simulated the sleep expert's approach by a noise detection algorithm. This research outlined five neural network models: four auxiliary models for each signal channel, and one primary model for all signal channels. The results of all of the models were weighted and integrated during prediction of sleep stages. Then an algorithm was designed to detect abnormal signals. When an electrode falling off was detected, the weights of the model corresponding to that electrode and the primary model were reset to 0 in the multi-model integration. As a result, the prediction was only obtained through the signal channels that did not fall off. Specifically, the integration method involved weighing the probability output by the final Softmax layer of each model. The weight of the base model was 4.5, and the weight of the remaining models was 1.

The patient’s turning over during sleep monitoring may also cause violent fluctuations in the signal, which may cause all models to fail. The fall-off signal often illustrates irregular fluctuations, with values frequently dropping to zero or reaching a maximum. In this study, if more than half of the signal values were the same within 3 seconds, we considered this 30 second period of time as the fall-off period.
